# Supplementary material for: Devices and Methods for Dosimetry of Personalized Photodynamic Therapy of Tumors: A Review on Recent Trends
Source: Cancers (Basel). 2024 Jul 8;16(13):2484. doi: 10.3390/cancers16132484 (PMC11240380; doi:10.3390/cancers16132484)
Supplement: Supplementary file 1 [file cancers-16-02484-s001.zip › cancers-3061578-supplementary.pdf]

Numbers	Localization	Country	Year	References
28	Cervix	Russia	2022	Afanasiev, M. S., Dushkin, A. D., Grishacheva, T. G., Afanasiev, S. S., & Academician, A. V. K. (2022). Photodynamic therapy for early-stage cervical cancer treatment. Photodiagnosis and Photodynamic Therapy, 37, 102620.
22	Cervix	Korea	2016	Park, Y. K., & Park, C. H. (2016). Clinical efficacy of photodynamic therapy. Obstetrics & gynecology science, 59(6), 479-488.
11	Cervix	USA	1997	Monk A., Brewer C., Van Nostrand K., Bems M. Photodynamic therapy using topically applied dihematoporphyrin ether in the treatment of cervical intraepithelial neoplasia, Gynecol Oncol, 1997, Vol. 64(1), pp. 70-5.
195	Cervix	Russia	2011	Trushina O.I., Novikova E.G. Possibilities of PDT for secondary prevention of virus-associated cervical pre-cancer, Sibirskii onkologicheskii zhurnal, 2011, No. 3, p. 45. (in Russian).
105	Cervix	Japan	2005	Yamaguchi, S., Tsuda, H., Takemori, M., Nakata, S., Nishimura, S., Kawamura, N., ... & Nishimura, R. (2005). Photodynamic therapy for cervical intraepithelial neoplasia. Oncology, 69(2), 110-116.
59	Cervix	Korea	2013	Choi, M. C., Jung, S. G., Park, H., Lee, S. Y., Lee, C., Hwang, Y. Y., & Kim, S. J. (2013). Photodynamic therapy for management of cervical intraepithelial neoplasia II and III in young patients and obstetric outcomes. Lasers in Surgery and Medicine, 45(9), 564-572.
54	Cervix	China	2021	Ran, R., Wang, M., Li, X., & Liu, Q. (2021). A prospective study of photodynamic therapy for cervical squamous intraepithelial lesion. Photodiagnosis and photodynamic therapy, 34, 102185.
76	Cervix	Brazil	2019	Inada, N. M., Buzzá, H. H., Leite, M. F. M., Kurachi, C., Trujillo, J. R., de Castro, C. A., ... & Bagnato, V. S. (2019). Long term effectiveness of photodynamic therapy for CIN treatment. Pharmaceuticals, 12(3), 107.
112	Cervix	Belarus	2010	Istomin, Y. P., Lapzevich, T. P., Chalau, V. N., Shliakhtsin, S. V., & Trukhachova, T. V. (2010). Photodynamic therapy of cervical intraepithelial neoplasia grades II and III with Photolon®. Photodiagnosis and Photodynamic Therapy, 7(3), 144-151.

472	Cervix	China	2014	Tao, X. H., Guan, Y., Shao, D., Xue, W., Ye, F. S., Wang, M., & He, M. H. (2014). Efficacy and safety of photodynamic therapy for cervical intraepithelial neoplasia: a systemic review. Photodiagnosis and photodynamic therapy, 11(2), 104-112.
262	Cervix	Germany	2015	Hillemanns, P., Garcia, F., Petry, K. U., Dvorak, V., Sadovsky, O., Iversen, O. E., & Einstein, M. H. (2015). A randomized study of hexaminolevulinate photodynamic therapy in patients with cervical intraepithelial neoplasia 1/2. American journal of obstetrics and gynecology, 212(4), 465-e1.
24	Cervix	USA	1997	Monk, B. J., Brewer, C., VanNostrand, K., Berns, M. W., McCullough, J. L., Tadir, Y., & Manetta, A. (1997). Photodynamic therapy using topically applied dihematoporphyrin ether in the treatment of cervical intraepithelial neoplasia. Gynecologic oncology, 64(1), 70-75.
7	Cervix	Germany	1999	Hillemanns, P., Korell, M., Schmitt-Sody, M., Baumgartner, R., Beyer, W., Kimmig, R., ... & Hepp, H. (1999). Photodynamic therapy in women with cervical intraepithelial neoplasia using topically applied 5-aminolevulinic acid. International journal of cancer, 81(1), 34-38.
59	Cervix	Germany	2014	Hillemanns, P., Petry, K. U., Soergel, P., Collinet, P., Ardaens, K., Gallwas, J., ... & Dannecker, C. (2014). Efficacy and safety of hexaminolevulinate photodynamic therapy in patients with low-grade cervical intraepithelial neoplasia. Lasers in surgery and medicine, 46(6), 456-461.
72	Cervix	Russia	2008	Trushina, O. I., Novikova, E. G., Sokolov, V. V., Filonenko, E. V., Chissov, V. I., & Vorozhtsov, G. N. (2008). Photodynamic therapy of virus-associated precancer and early stages cancer of cervix uteri. Photodiagnosis and Photodynamic Therapy, 5(4), 256-259.
52	Cervix	Russia	2022	Gilyadova, A., Ishchenko, A., Shiryaev, A., Alekseeva, P., Efendiev, K., Karpova, R., ... & Reshetov, I. (2022). Phototheranostics of cervical neoplasms with chlorin e6 photosensitizer. Cancers, 14(1), 211.
10	Cervix	Russia	2020	Alekseeva, P. M., Efendiev, K. T., Loshchenov, M. V., Shiryaev, A. A., Ishchenko, A. A., Gilyadova, A. V., ... & Loschenov, V. (2020). Combined spectral-and video-fluorescent diagnostics of cervical neoplasms for photodynamic therapy. Laser Physics Letters, 17(10), 105602.

45	Cervix	Russia	2022	Gilyadova, A., Ishchenko, A., Ishenko, A., Samoilova, S., Shiryaev, A., Kiseleva, A., ... & Reshetov, I. (2022). Analysis of the results of severe intraepithelial squamous cell lesions and preinvasive cervical cancer phototheranostics in women of reproductive age. Biomedicines, 10(10), 2521.
7	Cervix	Russia	2021	Efendiev, K. T., Alekseeva, P. M., Bikmukhametova, I. R., Piterskova, L. S., Orudzhova, K. F., Agabekova, U. D., ... & Loschenov, V. B. (2021). Comparative investigation of 5-aminolevulinic acid and hexyl aminolevulinate-mediated photodynamic diagnostics and therapy of cervical dysplasia and vulvar leukoplakia. Laser Physics Letters, 18(6), 065601.
40	Cervix	USA	2002	Keefe, K. A., Tadir, Y., Tromberg, B., Berns, M., Osann, K., Hashad, R., & Monk, B. J. (2002). Photodynamic therapy of high-grade cervical intraepithelial neoplasia with 5-aminolevulinic acid. Lasers in Surgery and Medicine: The Official Journal of the American Society for Laser Medicine and Surgery, 31(4), 289-293.
21	Cervix	Korea	2014	Choi, M. C., Jung, S. G., Park, H., Lee, S. Y., Lee, C., Hwang, Y. Y., & Kim, S. J. (2014). Fertility preservation by photodynamic therapy combined with conization in young patients with early stage cervical cancer: a pilot study. Photodiagnosis and photodynamic therapy, 11(3), 420-425.
13	Cervix	Brazil	2020	Vendette, A. C. F., Piva, H. L., Muehlmann, L. A., de Souza, D. A., Tedesco, A. C., & Azevedo, R. B. (2020). Clinical treatment of intra-epithelia cervical neoplasia with photodynamic therapy. International Journal of Hyperthermia, 37(3), 50-58.
292	Cervix	China	2018	Zhang, W., Zhang, A., Sun, W., Yue, Y., & Li, H. (2018). Efficacy and safety of photodynamic therapy for cervical intraepithelial neoplasia and human papilloma virus infection: A systematic review and meta-analysis of randomized clinical trials. Medicine, 97(21).
32	Cervix	USA	2013	Godoy, H., Vaddadi, P., Cooper, M., Frederick, P. J., Odunsi, K., & Lele, S. (2013). Photodynamic therapy effectively palliates gynecologic malignancies. European Journal of Gynaecological Oncology, 34(4), 300-302.

31	Cervix	China	2021	Wu, A., Li, Q., Ling, J., Gu, L., Hong, Z., Di, W., & Qiu, L. (2021). Effectiveness of photodynamic therapy in women of reproductive age with cervical high-grade squamous intraepithelial lesions (HSIL/CIN2). Photodiagnosis and photodynamic therapy, 36, 102517.
210	Cervix	China	2021	Ma, L., Gao, X., Geng, L., You, K., Wu, Z., Li, Y., ... & Guo, H. (2021). Efficacy and safety of photodynamic therapy mediated by 5-aminolevulinic acid for the treatment of cervical intraepithelial neoplasia 2: A single-center, prospective, cohort study. Photodiagnosis and photodynamic therapy, 36, 102472.
57	Cervix	China	2021	Cang, W., Gu, L., Hong, Z., Wu, A., Di, W., & Qiu, L. (2021). Effectiveness of photodynamic therapy with 5-aminolevulinic acid on HPV clearance in women without cervical lesions. Photodiagnosis and photodynamic therapy, 34, 102293.
13	Cervix	Austria	1996	Koren, H., & Alth, G. (1996). Photodynamic therapy in gynaecologic cancer. Journal of Photochemistry and Photobiology B: Biology, 36(2), 189-191.
67	Cervix	Germany	2012	Soergel, P., Dahl, G. F., Onsrud, M., & Hillemanns, P. (2012). Photodynamic therapy of cervical intraepithelial neoplasia 1–3 and human papilloma virus (HPV) infection with methylaminolevulinate and hexaminolevulinate—A double-blind, dose-finding study. Lasers in surgery and medicine, 44(6), 468-474.
48	Cervix	China	2022	Yuehui, S. U., Zhang, Y., Tong, Y., Zhang, L., Li, P., Zhang, H., ... & Zhang, T. (2022). Effect and rational application of topical photodynamic therapy (PDT) with 5-aminolevulinic acid (5-ALA) for treatment of cervical intraepithelial neoplasia with vaginal intraepithelial neoplasia. Photodiagnosis and photodynamic therapy, 37, 102634.
80	Cervix	China	2020	Li, D., Zhang, F., Shi, L., Lin, L., Cai, Q., & Xu, Y. (2020). Treatment of HPV infection-associated low grade cervical intraepithelial neoplasia with 5-aminolevulinic acid-mediated photodynamic therapy. Photodiagnosis and Photodynamic Therapy, 32, 101974.
10	Cervix	Poland	2019	Maździarz, A. (2019). Successful pregnancy and delivery following selective use of photodynamic therapy in treatment of cervix and vulvar diseases. Photodiagnosis and photodynamic therapy, 28, 65-68.

5	Cervix	China	2010	Wang, J., Xu, J., Chen, J., He, Q., Xiang, L., Huang, X., ... & Xu, S. (2010). Successful photodynamic therapy with topical 5-aminolevulinic acid for five cases of cervical intraepithelial neoplasia. Archives of gynecology and obstetrics, 282, 307-312.
183	Cervix	China	2022	Wang, B., Su, Y., Zhang, C., Zhou, M., Yuan, S., Zhang, M., ... & Zhang, T. (2022). The effect of local photodynamic therapy with 5-aminolevulinic acid in treating different grades of cervical intraepithelial neoplasia. Photodiagnosis and Photodynamic Therapy, 40, 103196.
8	Cervix	Japan	1993	Kato, H., Horai, T., Furuse, K., Fukuoka, M., Suzuki, S., Hiki, Y., ... & Hayata, Y. (1993). Photodynamic therapy for cancers: a clinical trial of porfimer sodium in Japan. Japanese journal of cancer research, 84(11), 1209-1214.
12	Cervix	UK	2003	Barnett, A. A., Haller, J. C., Cairnduff, F., Lane, G., Brown, S. B., & Roberts, D. J. (2003). A randomised, double-blind, placebo-controlled trial of photodynamic therapy using 5-aminolaevulinic acid for the treatment of cervical intraepithelial neoplasia. International journal of cancer, 103(6), 829-832.
56	Cervix	Japan	1996	Muroya, T., Suehiro, Y., Umayahara, K., Akiya, T., Iwabuchi, H., Sakunaga, H., ... & Tenjin, Y. (1996). Photodynamic therapy (PDT) for early cervical cancer. Gan to Kagaku ryoho. Cancer & Chemotherapy, 23(1), 47-56.
74	Cervix	Russia	2022	Ivanova, V. A., Nikitina, V. P., Verenikina, E. V., Zhenilo, O. E., & Ardzha, A. Y. (2022). Photodynamic therapy for early cervical cancer.
258	Cervix	China	2021	Gu, L., Cheng, M., Hong, Z., Di, W., & Qiu, L. (2021). The effect of local photodynamic therapy with 5-aminolevulinic acid for the treatment of cervical low-grade squamous intraepithelial lesions with high-risk HPV infection: A retrospective study. Photodiagnosis and photodynamic therapy, 33, 102172.
76	Cervix	China	2016	Fu, Y., Bao, Y., Hui, Y., Gao, X., Yang, M., & Chang, J. (2016). Topical photodynamic therapy with 5-aminolevulinic acid for cervical high-risk HPV infection. Photodiagnosis and photodynamic therapy, 13, 29-33.

110	Cervix	China	2016	Liu, Z., Zheng, H., Chen, X., & Qi, N. (2016). Comparison of the efficacy of ALA and high-frequency electric ion operating on cervical intraepithelial neoplasia grade I. Int. J. Clin. Exp. Med, 9, 16782-6.
51	Cervix	Japan	2020	Mizuno, M., Mitsui, H., Kajiyama, H., Teshigawara, T., Inoue, K., Takahashi, K., ... & Kikkawa, F. (2020). Efficacy of 5-aminolevulinic acid and LED photodynamic therapy in cervical intraepithelial neoplasia: A clinical trial. Photodiagnosis and Photodynamic Therapy, 32, 102004.
115	Cervix	China	2022	Chen, Y., Xu, Y., Zhang, Z., Xiong, Z., & Wu, D. (2022). 5-aminolevulinic acid-mediated photodynamic therapy effectively ameliorates HPV-infected cervical intraepithelial neoplasia. American Journal of Translational Research, 14(4), 2443.
83	Cervix	China	2022	Zhang, Y., Su, Y., Tang, Y., Qin, L., Shen, Y., Wang, B., ... & Zhang, T. (2022). Management of patients with positive margin after conization for high-grade cervical intraepithelial lesions. Lasers in Surgery and Medicine, 54(8), 1099-1106.
35	Cervix	USA	2016	Mayor, P. C., & Lele, S. (2016). Photodynamic therapy in gynecologic malignancies: a review of the Roswell Park Cancer Institute experience. Cancers, 8(10), 88.
25	Cervix	Germany	2010	Soergel, P., Loehr-Schulz, R., Hillemanns, M., Landwehr, S., Makowski, L., & Hillemanns, P. (2010). Effects of photodynamic therapy using topical applied hexylaminolevulinate and methylaminolevulinate upon the integrity of cervical epithelium. Lasers in surgery and medicine, 42(9), 784-790.
31	Cervix	Japan	2003	Ichimura, H., Yamaguchi, S., Kojima, A., Tanaka, T., Niiya, K., Takemori, M., ... & Nishimura, R. (2003). Eradication and reinfection of human papillomavirus after photodynamic therapy for cervical intraepithelial neoplasia. International journal of clinical oncology, 8, 322-325.
57	Skin	Italy	1995	Calzavara-Pinton, P. G. (1995). Repetitive photodynamic therapy with topical δ-aminolaevulinic acid as an appropriate approach to the routine treatment of superficial non-melanoma skin tumours. Journal of Photochemistry and Photobiology B: Biology, 29(1), 53-57.

95	Skin	Austria	1998	Fink-Puches, R., Soyer, H. P., Hofer, A., Kerl, H., & Wolf, P. (1998). Long-term follow-up and histological changes of superficial nonmelanoma skin cancers treated with topical δ-aminolevulinic acid photodynamic therapy. Archives of dermatology, 134(7), 821-826.
26	Skin	Germany	2000	Haller, J. C., Cairnduff, F., Slack, G., Schofield, J., Whitehurst, C., Tunstall, R., ... & Roberts, D. J. H. (2000). Routine double treatments of superficial basal cell carcinomas using aminolaevulinic acid-based photodynamic therapy. British Journal of Dermatology, 143(6), 1270-1275.
73	Skin	UK	2001	Morton, C. A., Whitehurst, C., McColl, J. H., Moore, J. V., & MacKie, R. M. (2001). Photodynamic therapy for large or multiple patches of Bowen disease and basal cell carcinoma. Archives of dermatology, 137(3), 319-324.
22	Skin	Norway	2000	Soler, A. M., Angell-Petersen, E., Warloe, T., Tausjø, J., Steen, H. B., Moan, J., & Giercksky, K. E. (2000). Photodynamic therapy of superficial basal cell carcinoma with 5-aminolevulinic acid with dimethylsulfoxide and ethylendiaminetetraacetic acid: a comparison of two light sources. Photochemistry and photobiology, 71(6), 724-729.
350	Skin	Norway	2001	Soler, A. M., Warloe, T., Berner, A., & Giercksky, A. K. (2001). A follow-up study of recurrence and cosmesis in completely responding superficial and nodular basal cell carcinomas treated with methyl 5-aminolaevulinate-based photodynamic therapy alone and with prior curettage. British Journal of Dermatology, 145(3), 467-471.
80	Skin	Sweden	1994	Svanberg, K., Andersson, T., Killander, D., Wang, I., Stenram, U., ANDERSSON-ENGELS, S., ... & Svanberg, S. (1994). Photodynamic therapy of non-melanoma malignant tumours of the skin using topical δ-amino levulinic acid sensitization and laser irradiation. British Journal of Dermatology, 130(6), 743-751.
24	Skin	Netherlands	2000	Thissen, M. R. T. M., Schroeter, C. A., & Neumann, H. A. M. (2000). SHORT COMMUNICATIONPhotodynamic therapy with delta-aminolaevulinic acid for nodular basal cell carcinomas using a prior debulking technique. British Journal of Dermatology, 142(2), 338-339.

699	Skin	Germany	2005	Szeimies, R. M., Karrer, S., Radakovic-Fijan, S., & Tanew, A. (2005, March). Photodynamic therapy using topical methyl aminolevulinate (MAL-PDT) compared with cryotherapy for actinic keratosis: A European prospective, randomized study. In JOURNAL OF THE AMERICAN ACADEMY OF DERMATOLOGY (Vol. 52, No. 3, pp. P164-P164). 11830 WESTLINE INDUSTRIAL DR, ST LOUIS, MO 63146-3318 USA: MOSBY, INC.
127	Skin	UK	2001	Varma, S., Wilson, H., Kurwa, H. A., Gambles, B., Charman, C., Pearse, A. D., ... & Anstey, A. V. (2001). Bowen’s disease, solar keratoses and superficial basal cell carcinomas treated by photodynamic therapy using a large-field incoherent light source. British Journal of Dermatology, 144(3), 567-574.
36	Skin	UK	1994	Cairnduff F, Stringer MR, Hudson EJ, et al. Superficial photodynamic therapy with topical 5-aminolaevulinic acid for superficial primary and secondary skin cancer. Br J Cancer 1994; 69: 605–8.
33	Skin	Austria	1998	Fink-Puches, R., Soyer, H. P., Hofer, A., Kerl, H., & Wolf, P. (1998). Long-term follow-up and histological changes of superficial nonmelanoma skin cancers treated with topical δ-aminolevulinic acid photodynamic therapy. Archives of dermatology, 134(7), 821-826.
40	Skin	UK	1996	Morton CA, Whitehurst C, Moseley H, et al. Comparison of photodynamic therapy with cryotherapy in the treatment of Bowen's disease. Br J Dermatol 1996; 135: 766–71.
61	Skin	UK	2000	Morton CA, Whitehurst C, Moore JV, et al. Comparison of red and green light in the treatment of Bowen's disease by photodynamic therapy. Br J Dermatol 2000; 143: 767–72.
85	Skin	UK	2001	Morton CA, Whitehurst C, McColl JH, et al. Photodynamic therapy for large or multiple patches of Bowen disease and basal cell carcinoma. Arch Dermatol 2001; 137: 319–24.

80	Skin	UK	2001	Varma S, Wilson H, Kurwa HA, et al. Bowen's disease, solar keratoses and superficial basal cell carcinomas treated by photodynamic therapy using a large-field incoherent light source. Br J Dermatol 2001; 144: 567–74.
196	Skin	Germany	2008	Szeimies, R. M., Ibbotson, S., Murrell, D. F., Rubel, D., Frambach, Y., De Berker, D., ... & Excilight Study Group. (2008). A clinical study comparing methyl aminolevulinate photodynamic therapy and surgery in small superficial basal cell carcinoma (8–20 mm), with a 12-month follow-up. Journal of the European Academy of Dermatology and Venereology, 22(11), 1302-1311.
243	Skin	USA	2004	Piacquadio, D. J., Chen, D. M., Farber, H. F., Fowler Jr, J. F., Glazer, S. D., Goodman, J. J., ... & Weinstein, G. D. (2004). Photodynamic Therapy With Aminolevulinic Acid Topical Solution andVisible Blue Light in the Treatment of Multiple Actinic Keratoses of the Faceand Scalp: Investigator-Blinded, Phase 3, Multicenter Trials. Archives of dermatology, 140(1), 41-46.
110	Skin	USA	2006	Tschen, E.H.; Wong, D.S.; Pariser, D.M.; Dunlap, F.E.; Houlihan, A.; Ferdon, M.B.; Bruce, S.; Jarratt, M.T.; Loss, R.W.; Weiss, J.; et al. Photodynamic therapy using aminolaevulinic acid for patients with nonhyperkeratotic actinic keratoses of the face and scalp: Phase IV multicentre clinical trial with 12-month follow up. Br. J. Dermatol. 2006, 155, 1262–1269.
46	Skin	UK	2016	Pariser, D. M., Houlihan, A., Ferdon, M. B., & Berg, J. E. (2016). Randomized vehicle-controlled study of short drug incubation aminolevulinic acid photodynamic therapy for actinic keratoses of the face or scalp. Dermatologic Surgery, 42(3), 296.
42	Skin	UK	2003	Pariser, D.M.; Lowe, N.J.; Stewart, D.M.; Jarratt, M.T.; Lucky, A.W.; Pariser, R.J.; Yamauchi, P.S. Photodynamic therapy with topical methyl aminolevulinate for actinic keratosis: Results of a prospective randomized multicenter trial. J. Am. Acad. Dermatol. 2003, 48, 227–232. [

211	Skin	Sweden	2005	Tarstedt, M.; Rosdahl, I.; Berne, B.; Svanberg, K.; Wennberg, A.M. A randomized multicenter study to compare two treatment regimens of topical methyl aminolevulinate (Metvix®)-PDT in actinic keratosis of the face and scalp. Acta Derm. Venereol. 2005, 85, 424–428.
99	Skin	UK	2015	Collier, N.J.; Ali, F.R.; Lear, J.T. No age-related decline in efficacy of photodynamic therapy for treatment of basal cell carcinoma. Br. J. Dermatol. 2015, 173, 1564–1565.
44	Skin	Norway	2012	Christensen, E.; Mørk, C.; Skogvoll, E. High and sustained efficacy after two sessions of topical 5-aminolaevulinic acid photodynamic therapy for basal cell carcinoma: A prospective, clinical and histological 10-year follow-up study. Br. J. Dermatol. 2012, 166, 1342–1348.
32	Skin	Romania	2013	Cosgarea, R.; Susan, M.; Crisan, M.; Senila, S. Photodynamic therapy using topical 5-aminolaevulinic acid vs. surgery for basal cell carcinoma. J. Eur. Acad. Dermatol. Venereol. 2013, 27, 980–984.
135	Skin	Italy	2011	Fantini, F.; Greco, A.D.G.C.; Del Giovane, C.; Cesinaro, A.M.; Venturini, M.; Zane, C.; Surrenti, T.; Peris, K.; Calzavara-Pinton, P.G. Photodynamic therapy for basal cell carcinoma: Clinical and pathological determinants of response. J. Eur. Acad. Dermatol. Venereol. 2011, 25, 896–901.
323	Skin	Netherlands	2016	Kessels, J.; Hendriks, J.; Nelemans, P.; Mosterd, K.; Kelleners-Smeets, N. Two-fold illumination in topical 5-aminolevulinic acid (ALA)-mediated photodynamic therapy (PDT) for superficial basal cell carcinoma (sBCC): A retrospective case series and cohort study. J. Am. Acad. Dermatol. 2016, 74, 899–906.
90	Skin	Denmark	2012	Lindberg-Larsen, R.; Sølvsten, H.; Kragbale, K. Evaluation of recurrence after photodynamic therapy with topical methylaminolaevulinate for 157 basal cell carcinomas in 90 patients. Acta Derm. Venereol. 2012, 92, 144–147. [
50	Skin	UK	2007	Rhodes, L.E.; de Rie, M.; Enstrom, Y.; Groves, R.; Morken, T.; Goulden, V.; Wong, G.A.; Grob, J.J.; Varma, S.; Wolf, P. Photodynamic therapy using topical methyl aminolevulinate vs. surgery for nodular basal cell carcinoma: Results of a multicenter randomized prospective trial. Arch. Dermatol. 2007, 143, 1131–1136.

85	Skin	Netherlands	2013	Roozeboom, M.H.; Aardoom, M.A.; Nelemans, P.J.; Thissen, M.R.T.M.; Kelleners-Smeets, N.W.J.; Kuijpers, D.I.M.; Mosterd, K. Fractionated 5-aminolevulinic acid photodynamic therapy after partial debulking versus surgical excision for nodular basal cell carcinoma: A randomized controlled trial with at least 5-year follow-up. J. Am. Acad. Dermatol. 2013, 69, 280–287.
67	Skin	Portugal	2015	Cabete, J.; Rafael, M.; Cravo, M.; Moura, C.; Sachse, F.; Pecegueiro, M. Long-term recurrence of nonmelanoma skin cancer after topical methylaminolevulinate photodynamic therapy in a dermato-oncology department. An. Bras. Dermatol. 2015, 90, 846–850.
96	Skin	UK	2006	Morton, C.; Horn, M.; Leman, J.; Tack, B.; Bedane, C.; Tjioe, M.; Ibbotson, S.; Khemis, A.; Wolf, P. Comparison of topical methyl aminolevulinate photodynamic therapy with cryotherapy or fluorouracil for treatment of squamous cell carcinoma in situ—Results of a multicenter randomized trial. Arch. Dermatol. 2006, 142, 729–735.
116	Skin	Sweden	2016	Tarstedt, M.; Gillstedt, M.; Wennberg Larkö, A.-M.; Paoli, J. Aminolevulinic acid and methyl aminolevulinate equally effective in topical photodynamic therapy for non-melanoma skin cancers. J. Eur. Acad. Dermatol. Venereol. 2016, 30, 420–423.
19	Skin	Russia	2022	Efendiev K., Alekseeva P. M., Shiryaev A. O., Skobeltsin A. S., Solonina I. L., Fatyanova A. S., Reshetov I. V., Loschenov V. B. Preliminary low-dose photodynamic exposure to the skin cancer with chlorin e6 photosensitizer. // Photodiagnosis and Photodynamic Therapy. 2022. Vol. 38. p. 102894.
4	Skin	Russia	2023	Efendiev K., Alekseeva P., Shiryaev A. O., Linkov K., Pisareva T., Reshetov I. V., Loschenov V. Near-infrared phototheranostics of tumors with protoporphyrin IX and chlorin e6 photosensitizers // Photodiagnosis and Photodynamic Therapy. 2023. Vol. 42. p. 103566.
10	Skin	Russia	2024	Efendiev K., Alekseeva P., Linkov K., Shiryaev A., Pisareva T., Gilyadova A., Reshetov I., Voitova A., Loschenov V. Tumor fluorescence and oxygenation monitoring during photodynamic therapy with chlorin e6 photosensitizer // Photodiagnosis and Photodynamic Therapy. 2024. p. 103969.

255	Skin	Russia	2016	Странадко, Е. Ф., Малова, Т. И., Волгин, В. Н., & Рябов, М. В. (2016). Лазерная фотодинамическая терапия-новая медицинская технология лечения рака кожи «неудобных» критических локализаций. Лазерная медицина, 20(4), 5-8.
53	Skin	Russia	2006	Stranadko E.F., Ryabov M.V. Photodynamic therapy of skin cancer with Photolon: experience of application and adjustment of parameters, Lazernaya meditsina, 2006, Vol. 10, No. 2, pp. 4-10. (in Russian).
334	Skin	Russia	2014	Kapinus V.N., Kaplan M.A., Spichenkova I.S., Shubina A.M., Yaroslavtseva-Isaeva E.V. Photodynamic therapy of skin malignant neoplasms, Fotodinamicheskaya terapiya i fotodiagnostika, 2014, No. 3, pp. 9-14. (in Russian).
91	Skin	Russia	2011	Волгин., Эффективность фотодинамической терапии базально-клеточного рака кожи с фотосенсом и оценка отдаленных результатов лечения, 2011.
36	Skin	Russia	2011	Kaplan M.A., Kapinus V.N., Goranskaya E.V. Photodynamic therapy of intradermal metastases of breast cancer, Opukholi zhenskoi reproduktivnoi sistemy, 2011, No. 4, pp. 28-31. (in Russian).
21	Skin	Russia	2012	Filonenko E.V., Okushko A.N., Sukhin D.G., Yanikova A.G. Photody-namic therapy of patients with intradermal melanoma metastases, Onkologiya. Zhurnal im. P.A. Gertsena, 2012, No. 3, pp. 52-54. (in Russian).
96	Skin	Russia	2015	Волгин, В. Н., Странадко, Е. Ф., & Кагоянц, Р. В. (2014). Новый метод лечения базально-клеточного рака кожи ЛОР-органов с применением фотодинамической терапии. Российские медицинские вести, 19(3), 75-80.
26	Skin	Russia	2012	Странадко, Е. Ф., Титова, В. А., & Петровский, В. Ю. (2012). Фотодинамическая терапия: полимодальные программы лечения рака различных локализаций. Лазерная медицина, 16(3), 4-7.
35	Head and neck	Japan	2018	Hosokawa, S., Takebayashi, S., Takahashi, G., Okamura, J., & Mineta, H. (2018). Photodynamic therapy in patients with head and neck squamous cell carcinoma. Lasers in Surgery and Medicine, 50(5), 420-426.
30	Head and neck	Germany	1990	Feyh, J., Goetz, A., Müller, W., Königsberger, R., & Kastenbauer, E. (1990). Photodynamic therapy in head and neck surgery. Journal of Photochemistry and Photobiology B: Biology, 7(2-4), 353-358.

12	Head and neck	USA	1990	Wenig BL, Kurtzman DM, Grossweiner L, et al. Photodynamic therapy in the treatment of squamous cell carcinoma of the head and neck. Arch Otolaryngol Head Neck Surg 1990; 116: 126770.
32	Head and neck	France	1990	Freche C, DeCorbiere S. Use of photodynamic therapy in the treatment of vocal cord carcinoma. J Photochem Photobiol 1990; 6: 2916.
11	Head and neck	UK	1994	Grant W, Hopper C, Speight PM, Macrobert AJ, Bown SG. Photodynamic therapy of malignant and premalignant lesions in patients with field cancerization of the oral cavity. J Laryngol Otol 1994; 93(107): 1140–5.
65	Head and neck	USA	1995	Biel MA. Photodynamic therapy of head and neck cancers. Semin Surg Oncol 1995; 11: 3559.
30	Head and neck	USA	2009	Rigual, N. R., Thankappan, K., Cooper, M., Sullivan, M. A., Dougherty, T., Popat, S. R., ... & Henderson, B. (2009). Photodynamic therapy for head and neck dysplasia and cancer. Archives of otolaryngology–head & neck surgery, 135(8), 784-788.
15	Head and neck	USA	2013	Rigual, N. R., Shafirstein, G., Frustino, J., Seshadri, M., Cooper, M., Wilding, G., ... & Henderson, B. (2013). Adjuvant intraoperative photodynamic therapy in head and neck cancer. JAMA otolaryngology–head & neck surgery, 139(7), 706-711.
45	Head and neck	UK	2004	Lou P. J. et al. Interstitial photodynamic therapy as salvage treatment for recurrent head and neck cancer //British Journal of Cancer. – 2004. – Т. 91. – №. 3. – С. 441-446.
27	Head and neck	Netherlands	2007	Copper, M. P., Triesscheijn, M., Tan, I. B., Ruevekamp, M. C., & Stewart, F. A. (2007). Photodynamic therapy in the treatment of multiple primary tumours in the head and neck, located to the oral cavity and oropharynx. Clinical otolaryngology, 32(3), 185-189.
12	Head and neck	USA	1990	Schweitzer, V. G. (1990). Photodynamic therapy for treatment of head and neck cancer. Otolaryngology–Head and Neck Surgery, 102(3), 225-232.

29	Lungs	Japan	1993	Kato, H., Horai, T., Furuse, K., Fukuoka, M., Suzuki, S., Hiki, Y., ... & Hayata, Y. (1993). Photodynamic therapy for cancers: a clinical trial of porfimer sodium in Japan. Japanese journal of cancer research, 84(11), 1209-1214.
16	Head and neck	Russia	2021	Alekseeva, P. M., Efendiev, K. T., Shiryaev, A. A., Rusakov, M. A., Simonova, M. S., Samoylova, S. I., ... & Loschenov, V. B. (2021). Sublingual administration of 5-aminolevulinic acid for laser-induced photodiagnostics and photodynamic therapy of oral cavity and larynx cancers. Photodiagnosis and Photodynamic Therapy, 34, 102289.
12	Stomach	Japan	1993	Kato, H., Horai, T., Furuse, K., Fukuoka, M., Suzuki, S., Hiki, Y., ... & Hayata, Y. (1993). Photodynamic therapy for cancers: a clinical trial of porfimer sodium in Japan. Japanese journal of cancer research, 84(11), 1209-1214.
5	Brain	Russia	2021	Kustov DM, Kozlikina EI, Efendiev KT, Loshchenov MV, Grachev PV, Maklygina YS, Trifonov IS, Baranov AV, Stranadko EF, Panchenkov DN, Krylov VV, Loschenov VB. Laser-induced fluorescent visualization and photodynamic therapy in surgical treatment of glial brain tumors. Biomed Opt Express. 2021 Mar 1;12(3):1761-1773. doi: 10.1364/BOE.415936. PMID: 33796385; PMCID: PMC7984776.
39	Head and neck	Netherlands	2010	Tan, I. B., Dolivet, G., Ceruse, P., Poorten, V. V., Roest, G., & Rauschning, W. (2010). Temoporfin-mediated photodynamic therapy in patients with advanced, incurable head and neck cancer: A multicenter study. Head & neck, 32(12), 1597-1604.
25	Head and neck	Netherlands	2003	Copper, M. P., Tan, I. B., Oppelaar, H., Ruevekamp, M. C., & Stewart, F. A. (2003). Meta-tetra (hydroxyphenyl) chlorin photodynamic therapy in early-stage squamous cell carcinoma of the head and neck. Archives of Otolaryngology–Head & Neck Surgery, 129(7), 709-711.
5	Head and neck	India	2015	Selvam NP, Sadaksharam J, Singaravelu G, et al. Treatment of oral leukoplakia with photodynamic therapy: a pilot study. J Can Res Ther. 2015; 11: 464-467

30	Cervix	Mexico	2017	Maldonado Alvarado, E., Osorio Peralta, M. O., Moreno Vázquez, A., Martínez Guzmán, L. A., Melo Petrone, M. E., Enriquez Mar, Z. I., ... & Ramon Gallegos, E. (2017). Effectiveness of photodynamic therapy in elimination of HPV-16 and HPV-18 associated with CIN I in Mexican women. Photochemistry and photobiology, 93(5), 1269-1275.
23	Head and neck	Poland	2014	Pietruska M, Sobaniec S, Bernaczyk P, et al. Clinical evaluation of photodynamic therapy efficacy in the treatment of oral leukoplakia. Photodiagnosis Photodyn Ther. 2014; 11: 34-40.
85	Head and neck	Poland	2012	Kawczyk-Krupka A, Waskowska J, Raczkowska-Siostrzonek A, et al. Comparison of cryotherapy and photodynamic therapy in treatment of oral leukoplakia. Photodiagn Photodyn Ther. 2012; 9: 148-155.
147	Head and neck	UK	2011	Jerjes W, Upile T, Hamdoon Z, et al. Photodynamic therapy outcome for oral dysplasia. Lasers Surg Med. 2011; 43: 192-199.
23	Head and neck	USA	2011	Shafirstein G, Friedman A, Siegel E, et al. Using 5-aminolevulinic acid and pulsed dye laser for photodynamic treatment of oral leukoplakia. Arch Otolaryngol Head Neck Surg. 2011; 137: 1117-1123.
80	Head and neck	China	2010	Lin HP, Chen MH, Yu HC, et al. Topical photodynamic therapy is very effective for oral verrucous hyperplasia and oral erythroleukoplakia. J Oral Pathol Med. 2010; 39: 624-630.
46	Head and neck	China	2009	Yu CH, Lin HP, Chen HM, et al. Comparison of clinical outcomes of oral erythroleukoplakia treated with photodynamic therapy using either light-emitting diode or laser light. Lasers Surg Med. 2009; 41: 628-633.
36	Head and neck	China	2008	Yu CH, Chen HM, Hung HY, et al. Photodynamic therapy outcome for oral verrucous hyperplasia depends on the clinical appearance, size, color, epithelial dysplasia, and surface keratin thickness of the lesion. Oral Oncol. 2008; 44: 595-600.

32	Head and neck	China	2005	Chen HM, Yu CH, Tu PC, et al. Successful treatment of oral verrucous hyperplasia and oral leukoplakia with topical 5-aminolevulinic acid-mediated photodynamict herapy. Lasers Surg Med. 2005; 37: 114-122.
5	Head and neck	China	2004	Chen HM, Chen CT, Yang H, et al. Successful treatment of oral verrucous hyperplasia with topical 5-aminolevulinic acid-mediated photodynamic therapy. Oral Oncol. 2004; 40: 630-637.
31	Head and neck	China	2004	Tsai JC, Chiang CP, Chen HM, et al. Photodynamic therapy of oral dysplasia with topical 5-aminolevulinic acid and light-emitting diode array. Lasers Surg Med. 2004; 34: 18-24.
12	Head and neck	Poland	2003	Sieron A, Adamek M, Kawczyk-Krupka A, et al. Photodynamic therapy (PDT) using topically applied delta-aminolevulinic acid (ALA) for the treatment of oral leukoplakia. J Oral Pathol Med. 2003; 32: 330-336.
5	Head and neck	Poland	2001	Sieron A, Namyslowski G, Misiolek M, et al. Photodynamic therapy of premalignant lesions and local recurrence of laryngeal and hypopharyngeal cancers. Eur Arch Otorhinolaryngol. 2001; 258: 349-352.
12	Head and neck	Germany	1998	Kubler A, Haase T, Rheinwald M, et al. Treatment of oral leukoplakia by topical application of 5-aminolevulinicacid. Int J Oral Maxillofac Surg. 1998; 27: 466-469.
12	Head and neck	UK	1996	Fan KF, Hopper C, Speight PM, et al. Photodynamic therapy using 5-aminolevulinic acid for premalignant and malignant lesions of the oral cavity. Cancer. 1996; 78: 1374-1383.
11	Head and neck	UK	1993	Grant WE, Hopper C, Speight PM, et al. Photodynamic therapy of malignant and premalignant lesions in patients with ‘field cancerization’ of the oral cavity. J Laryngol Otol. 1993; 107: 1140-1145.
114	Head and neck	UK	2004	Hopper C, Kubler A, Lewis H, et al. m-THPC-mediated photodynamic therapy for early oral squamous cell carcinoma. Int J Cancer. 2004a; 111: 138-146.

128	Head and neck	India	2004	D'Cruz AK, Robinson MH, Biel MA. mTHPC-mediated photodynamic therapy in patients with advanced, incurable head and neck cancer: a multicentre study of 128 patients. Head Neck. 2004; 26: 232-240.
20	Head and neck	USA	2001	Schweitzer VG. PHOTOFRIN-mediated photodynamic therapy for treatment of early stage oral cavity and laryngeal malignancies. Lasers Surg Med. 2001; 29: 305-313.
20	Head and neck	UK	1997	Fan KC, Hopper C, Speight PM, et al. Photodynamic therapy using mTHPC for malignant disease in the oral cavity. Int J Cancer. 1997; 73: 25-32.
35	Head and neck	USA	2016	Ahn, P. H., Quon, H., O'Malley, B. W., Weinstein, G., Chalian, A., Malloy, K., ... & Busch, T. M. (2016). Toxicities and early outcomes in a phase 1 trial of photodynamic therapy for premalignant and early stage head and neck tumors. Oral oncology, 55, 37-42.
5	Head and neck	Russia	2019	Farrakhova, D., Shiryaev, A., Yakovlev, D., Efendiev, K., Maklygina, Y., Borodkin, A., ... & Loschenov, V. (2019). Trials of a fluorescent endoscopic video system for diagnosis and treatment of the head and neck cancer. Journal of Clinical Medicine, 8(12), 2229.
26	Head and neck	Belgium	2021	Lambert, A., Nees, L., Nuyts, S., Clement, P., Meulemans, J., Delaere, P., & Vander Poorten, V. (2021). Photodynamic therapy as an alternative therapeutic tool in functionally inoperable oral and oropharyngeal carcinoma: a single tertiary center retrospective cohort analysis. Frontiers in Oncology, 11, 626394.
170	Head and neck	Netherlands	2011	Karakullukcu, B., van Oudenaarde, K., Copper, M. P., Klop, W. M. C., van Veen, R., Wildeman, M., & Bing Tan, I. (2011). Photodynamic therapy of early stage oral cavity and oropharynx neoplasms: an outcome analysis of 170 patients. European Archives of Oto-Rhino-Laryngology, 268, 281-288.

108	Head and neck	Brazil	2022	Fonseca, L. L., Durães, C. P., da Silva Menezes, A. S., Tabosa, A. T. L., Barbosa, C. U., de Paulo Santiago Filho, A., ... & Guimarães, A. L. S. (2022). Comparison between two antimicrobial photodynamic therapy protocols for oral candidiasis in patients undergoing treatment for head and neck cancer: A two-arm, single-blind clinical trial. Photodiagnosis and Photodynamic Therapy, 39, 102983.
2	Head and neck	Russia	2023	Panaseykin, Y. A., Kapinus, V. N., Filonenko, E. V., Polkin, V. V., Sevrukov, F. E., Isaev, P. A., ... & Kaprin, A. D. (2023). Photodynamic therapy treatment of oral cavity cancer in patients with comorbidities. Biomedical Photonics, 11(4), 19-24.
30	Head and neck	USA	2010	Schweitzer, V. G., & Somers, M. L. (2010). PHOTOFRIN-mediated photodynamic therapy for treatment of early stage (Tis-T2N0M0) SqCCa of oral cavity and oropharynx. Lasers in Surgery and Medicine: The Official Journal of the American Society for Laser Medicine and Surgery, 42(1), 1-8.
22	Brain	Japan	2016	Akimoto, J. (2016). Photodynamic therapy for malignant brain tumors. Neurologia medico-chirurgica, 56(4), 151-157.
112	Brain	Canada	2006	Muller, P. J., & Wilson, B. C. (2006). Photodynamic therapy of brain tumors—a work in progress. Lasers in Surgery and Medicine: The Official Journal of the American Society for Laser Medicine and Surgery, 38(5), 384-389.
22	Brain	USA	2004	Schmidt, M. H., Meyer, G. A., Reichert, K. W., Cheng, J., Krouwer, H. G., Ozker, K., & Whelan, H. T. (2004). Evaluation of photodynamic therapy near functional brain tissue in patients with recurrent brain tumors. Journal of neuro-oncology, 67, 201-207.
50	Brain	Canada	1990	Muller, P. J., & Wilson, B. C. (1990). Photodynamic therapy of malignant brain tumours. Lasers in Medical Science, 5, 245-252.
41	Brain	Spain	2015	Vanaclocha, V., Sureda, M., Azinovic, I., Rebollo, J., Cañón, R., Sapena, N. S., ... & Brugarolas, A. (2015). Photodynamic therapy in the treatment of brain tumours. A feasibility study. Photodiagnosis and Photodynamic Therapy, 12(3), 422-427.
358	Brain	Australia	2004	Stylli, S. S., Howes, M., MacGregor, L., Rajendra, P., & Kaye, A. H. (2004). Photodynamic therapy of brain tumours: evaluation of porphyrin uptake versus clinical outcome. Journal of Clinical Neuroscience, 11(6), 584-596.

18	Brain	USA	2000	Krishnamurthy, S., Powers, S. K., Witmer, P., & Brown, T. (2000). Optimal light dose for interstitial photodynamic therapy in treatment for malignant brain tumors. Lasers in Surgery and Medicine: The Official Journal of the American Society for Laser Medicine and Surgery, 27(3), 224-234.
56	Brain	Canada	1995	Muller, P. J., & Wilson, B. C. (1995, September). Photodynamic therapy for recurrent supratentorial gliomas. In Seminars in surgical oncology (Vol. 11, No. 5, pp. 346-354). New York: John Wiley & Sons, Inc..
27	Brain	Japan	2013	Muragaki, Y., Akimoto, J., Maruyama, T., Iseki, H., Ikuta, S., Nitta, M., ... & Kayama, T. (2013). Phase II clinical study on intraoperative photodynamic therapy with talaporfin sodium and semiconductor laser in patients with malignant brain tumors. Journal of neurosurgery, 119(4), 845-852.
20	Brain	Canada	1996	Muller, P. J., & Wilson, B. C. (1996). Photodynamic therapy for malignant newly diagnosed supratentorial gliomas. Journal of clinical laser medicine & surgery, 14(5), 263-270.
15	Brain	USA	1993	Origitano, T. C., & Reichman, O. H. (1993). Photodynamic therapy for intracranial neoplasms: development of an image-based computer-assisted protocol for photodynamic therapy of intracranial neoplasms. Neurosurgery, 32(4), 587-596.
51	Brain	Austria	1995	Kostron, H., Hochleitner, B. W., Obwegeser, A., & Seiwald, M. (1995, March). Clinical and experimental results of photodynamic therapy in neurosurgery. In 5th International Photodynamic Association Biennial Meeting (Vol. 2371, pp. 126-128). SPIE.
22	Brain	Austria	2001	Zimmermann, A., Ritsch-Marte, M., & Kostron, H. (2001). mTHPC-mediated Photodynamic Diagnosis of Malignant Brain Tumors¶. Photochemistry and photobiology, 74(4), 611-616.
14	Brain	UK	2009	Aziz, F., Telara, S., Moseley, H., Goodman, C., Manthri, P., & Eljamel, M. S. (2009). Photodynamic therapy adjuvant to surgery in metastatic carcinoma in brain. Photodiagnosis and Photodynamic Therapy, 6(3-4), 227-230.
10	Brain	France	2021	Vermandel, M., Dupont, C., Lecomte, F., Leroy, H. A., Tuleasca, C., Mordon, S., ... & Reyns, N. (2021). Standardized intraoperative 5-ALA photodynamic therapy for newly diagnosed glioblastoma patients: a preliminary analysis of the INDYGO clinical trial. Journal of neuro-oncology, 152, 501-514.

14	Brain	Japan	2012	Akimoto, J., Haraoka, J., & Aizawa, K. (2012). Preliminary clinical report on safety and efficacy of photodynamic therapy using talaporfin sodium for malignant gliomas. Photodiagnosis and photodynamic therapy, 9(2), 91-99.
47	Brain	Germany	2021	Lietke, S., Schmutzer, M., Schwartz, C., Weller, J., Siller, S., Aumiller, M., ... & Thon, N. (2021). Interstitial photodynamic therapy using 5-ALA for malignant glioma recurrences. Cancers, 13(8), 1767.
73	Brain	UK	2012	Lyons, M., Phang, I., & Eljamel, S. (2012). The effects of PDT in primary malignant brain tumours could be improved by intraoperative radiotherapy. Photodiagnosis and photodynamic therapy, 9(1), 40-45.
19	Brain	Japan	2018	Nitta, M., Muragaki, Y., Maruyama, T., Iseki, H., Komori, T., Ikuta, S., ... & Kawamata, T. (2018). Role of photodynamic therapy using talaporfin sodium and a semiconductor laser in patients with newly diagnosed glioblastoma. Journal of neurosurgery, 131(5), 1361-1368.
70	Brain	Japan	2022	Kobayashi, T., Nitta, M., Shimizu, K., Saito, T., Tsuzuki, S., Fukui, A., ... & Muragaki, Y. (2022). Therapeutic Options for Recurrent Glioblastoma—Efficacy of Talaporfin Sodium Mediated Photodynamic Therapy. Pharmaceutics, 14(2), 353.
3	Brain	Japan	2019	Akimoto, J., Fukami, S., Suda, T., Ichikawa, M., Haraoka, R., Kohno, M., ... & Kuroda, M. (2019). First autopsy analysis of the efficacy of intra-operative additional photodynamic therapy for patients with glioblastoma. Brain Tumor Pathology, 36, 144-151.
20	Brain	Germany	2020	Schipmann, S., Müther, M., Stögbauer, L., Zimmer, S., Brokinkel, B., Holling, M., ... & Stummer, W. (2020). Combination of ALA-induced fluorescence-guided resection and intraoperative open photodynamic therapy for recurrent glioblastoma: case series on a promising dual strategy for local tumor control. Journal of neurosurgery, 134(2), 426-436.
16	Bile ducts	Germany	2005	Zoepf, T., Jakobs, R., Arnold, J. C., Apel, D., & Riemann, J. F. (2005). Palliation of nonresectable bile duct cancer: improved survival after photodynamic therapy. Official journal of the American College of Gastroenterology| ACG, 100(11), 2426-2430.

23	Bile ducts	Austria	2004	Wiedmann, M., Berr, F., Schiefke, I., Witzigmann, H., Kohlhaw, K., Mössner, J., & Caca, K. (2004). Photodynamic therapy in patients with non-resectable hilar cholangiocarcinoma: 5-year follow-up of a prospective phase II study. Gastrointestinal Endoscopy, 60(1), 68-75.
33	Bile ducts	Russia	2019	Shiryaev, A. A., Musaev, G. K., Levkin, V. V., Reshetov, I. V., Loshchenov, M. V., Alekseeva, P. M., ... & Loschenov, V. B. (2019). Combined treatment of nonresectable cholangiocarcinoma complicated by obstructive jaundice. Photodiagnosis and photodynamic therapy, 26, 218-223.
14	Bile ducts	Russia	2017	Shiryaev, A. A., Musaev, G. K., Loshenov, M. V., Borodkin, A. V., Levkin, V. V., Okhotnikova, N. L., ... & Loshenov, V. B. (2017). Fluorescence diagnosis and photodynamic therapy in combined treatment of cholangiocarcinoma. Biomedical Photonics, 5(4), 15-24.
8	Bile ducts	Japan	2012	Nanashima, A., Abo, T., Nonaka, T., Nonaka, Y., Morisaki, T., Uehara, R., ... & Nagayasu, T. (2012). Photodynamic therapy using talaporfin sodium (Laserphyrin®) for bile duct carcinoma: A preliminary clinical trial. Anticancer research, 32(11), 4931-4938.
25	Bile ducts	Japan	2014	Nanashima, A., Isomoto, H., Abo, T., Nonaka, T., Morisaki, T., Arai, J., ... & Nagayasu, T. (2014). How to access photodynamic therapy for bile duct carcinoma. Annals of Translational Medicine, 2(3).
11	Bile ducts	Korea	2013	Bahng, S., Yoo, B. C., Paik, S. W., Koh, K. C., Lee, K. T., Lee, J. K., ... & Lee, K. H. (2013). Photodynamic therapy for bile duct invasion of hepatocellular carcinoma. Photochemical & Photobiological Sciences, 12, 439-445.
8	Bile ducts	Germany	2001	Zoepf, T., Jakobs, R., Arnold, J. C., Apel, D., Rosenbaum, A., & Riemann, J. F. (2001). Photodynamic therapy for palliation of nonresectable bile duct cancer—preliminary results with a new diode laser system. The American journal of gastroenterology, 96(7), 2093-2097.
13	Bile ducts	Germany	1998	Ortner, M. A. E., Liebetruth, J., Schreiber, S., Hanft, M., Wruck, U., Fusco, V., ... & Lochs, H. (1998). Photodynamic therapy of nonresectable cholangiocarcinoma. Gastroenterology, 114(3), 536-542.

21	Bile ducts	Austria	2013	Wagner, A., Kiesslich, T., Neureiter, D., Friesenbichler, P., Puespoek, A., Denzer, U. W., ... & Berr, F. (2013). Photodynamic therapy for hilar bile duct cancer: clinical evidence for improved tumoricidal tissue penetration by temoporfin. Photochemical & Photobiological Sciences, 12, 1065-1073.
29	Bile ducts	Austria	2015	Wagner, A., Denzer, U. W., Neureiter, D., Kiesslich, T., Puespoeck, A., Rauws, E. A., ... & Wolkersdörfer, G. W. (2015). Temoporfin improves efficacy of photodynamic therapy in advanced biliary tract carcinoma: A multicenter prospective phase II study. Hepatology, 62(5), 1456-1465.
2	Bile ducts	Japan	2020	Nanashima, A., Hiyoshi, M., Imamura, N., Hamada, T., Nishida, T., Kawakami, H., ... & Kai, M. (2020). Two cases of bile duct carcinoma patients who underwent the photodynamic therapy using talaporfin sodium (Laserphyrin®). Clinical Journal of Gastroenterology, 13, 102-109.
6	Bile ducts	California	2001	Rumalla, A., Baron, T. H., Wang, K. K., Gores, G. J., Stadheim, L. M., & De Groen, P. C. (2001). Endoscopic application of photodynamic therapy for cholangiocarcinoma. Gastrointestinal endoscopy, 53(4), 500-504.
24	Bile ducts	Korea	2005	Shim, C. S., Cheon, Y. K., Cha, S. W., Bhandari, S., Moon, J. H., Cho, Y. D., ... & Kim, B. S. (2005). Prospective study of the effectiveness of percutaneous transhepatic photodynamic therapy for advanced bile duct cancer and the role of intraductal ultrasonography in response assessment. Endoscopy, 37(05), 425-433.
5	Bile ducts	Japan	2004	Nanashima, A., Yamaguchi, H., Shibasaki, S., Ide, N., Sawai, T., Tsuji, T., ... & Nagayasu, T. (2004). Adjuvant photodynamic therapy for bile duct carcinoma after surgery: a preliminary study. Journal of gastroenterology, 39, 1095-1101.
8	Bile ducts	USA	2005	Harewood, G. C., Baron, T. H., Rumalla, A., Wang, K. K., Gores, G. J., Stadheim, L. M., & De Groen, P. C. (2005). Pilot study to assess patient outcomes following endoscopic application of photodynamic therapy for advanced cholangiocarcinoma. Journal of gastroenterology and hepatology, 20(3), 415-420.

72	Bile ducts	Korea	2012	Cheon, Y. K., Lee, T. Y., Lee, S. M., Yoon, J. Y., & Shim, C. S. (2012). Longterm outcome of photodynamic therapy compared with biliary stenting alone in patients with advanced hilar cholangiocarcinoma. HPB, 14(3), 185-193.
20	Bile ducts	Germany	2003	Ortner, M. E., Caca, K., Berr, F., Liebetruth, J., Mansmann, U., Huster, D., ... & Lochs, H. (2003). Successful photodynamic therapy for nonresectable cholangiocarcinoma: a randomized prospective study. Gastroenterology, 125(5), 1355-1363.
88	Bile ducts	Austria	2017	Dolak, W., Schwaighofer, H., Hellmich, B., Stadler, B., Spaun, G., Plieschnegger, W., ... & Austrian PDT Study Group. (2017). Photodynamic therapy with polyhematoporphyrin for malignant biliary obstruction: A nationwide retrospective study of 150 consecutive applications. United European gastroenterology journal, 5(1), 104-110.
21	Bile ducts	Germany	2001	Ortner, M. (2001). Photodynamic therapy for cholangiocarcinoma. Journal of hepato-biliary-pancreatic surgery, 8, 137-139.
74	Bile ducts	Korea	2014	Hong, M. J., Cheon, Y. K., Lee, E. J., Lee, T. Y., & Shim, C. S. (2014). Long-term outcome of photodynamic therapy with systemic chemotherapy compared to photodynamic therapy alone in patients with advanced hilar cholangiocarcinoma. Gut and liver, 8(3), 318.
19	Bile ducts	USA	2008	Kahaleh, M., Mishra, R., Shami, V. M., Northup, P. G., Berg, C. L., Bashlor, P., ... & Yeaton, P. (2008). Unresectable cholangiocarcinoma: comparison of survival in biliary stenting alone versus stenting with photodynamic therapy. Clinical Gastroenterology and Hepatology, 6(3), 290-297.
29	Bile ducts	Germany	2007	Dechene, A., Hilgard, P., Maldonado-Lopez, E. J., Riemann, J. F., Gerken, G., & Zoepf, T. (2007). Survival difference in patients with photodynamic therapy of nonresectable bile duct cancer using different hematoporphyrins. Gastrointestinal Endoscopy, 65(5), AB227.
7	Bile ducts	Austria	2003	Wiedmann, M., Caca, K., Berr, F., Schiefke, I., Tannapfel, A., Wittekind, C., ... & Witzigmann, H. (2003). Neoadjuvant photodynamic therapy as a new approach to treating hilar cholangiocarcinoma: a phase II pilot study. Cancer: Interdisciplinary International Journal of the American Cancer Society, 97(11), 2783-2790.

14	Bile ducts	France	2009	Fuks, D., Bartoli, E., Delcenserie, R., Yzet, T., Celice, P., Sabbagh, C., ... & Regimbeau, J. M. (2009). Biliary drainage, photodynamic therapy and chemotherapy for unresectable cholangiocarcinoma with jaundice. Journal of gastroenterology and hepatology, 24(11), 1745-1752.
24	Bile ducts	Germany	2003	Dumoulin, F. L., Gerhardt, T., Fuchs, S., Scheurlen, C., Neubrand, M., Layer, G., & Sauerbruch, T. (2003). Phase II study of photodynamic therapy and metal stent as palliative treatment for nonresectable hilar cholangiocarcinoma. Gastrointestinal endoscopy, 57(7), 860-867.
68	Bile ducts	Germany	2006	Witzigmann, H., Berr, F., Ringel, U., Caca, K., Uhlmann, D., Schoppmeyer, K., ... & Wiedmann, M. (2006). Surgical and palliative management and outcome in 184 patients with hilar cholangiocarcinoma: palliative photodynamic therapy plus stenting is comparable to r1/r2 resection. Annals of surgery, 244(2), 230.
23	Bile ducts	UK	2009	Quyn, A. J., Ziyaie, D., Polignano, F. M., & Tait, I. S. (2009). Photodynamic therapy is associated with an improvement in survival in patients with irresectable hilar cholangiocarcinoma. HPB, 11(7), 570-577.
4	Bile ducts	Japan	2004	Suzuki, S., Inaba, K., Yokoi, Y., Ohata, K., Ota, S., Azuma, M., ... & Nakamura, S. (2004). Photodynamic therapy for malignant biliary obstruction: a case series. Endoscopy, 36(01), 83-87.
25	Bile ducts	UK	2007	Prasad, G. A., Wang, K. K., Baron, T. H., Buttar, N. S., Wongkeesong, L. M., Roberts, L. R., ... & Borkenhagen, L. S. (2007). Factors associated with increased survival after photodynamic therapy for cholangiocarcinoma. Clinical Gastroenterology and Hepatology, 5(6), 743-748.
42	Bile ducts	UK	2011	Matull, W. R., Dhar, D. K., Ayaru, L., Sandanayake, N. S., Chapman, M. H., Dias, A., ... & Pereira, S. P. (2011). R0 but not R1/R2 resection is associated with better survival than palliative photodynamic therapy in biliary tract cancer. Liver International, 31(1), 99-107.

10	Bile ducts	Germany	2011	Höblinger, A., Gerhardt, T., Gonzalez-Carmona, M. A., Hüneburg, R., Sauerbruch, T., & Schmitz, V. (2011). Feasibility and safety of long-term photodynamic therapy (PDT) in the palliative treatment of patients with hilar cholangiocarcinoma. European journal of medical research, 16, 391-395.
37	Bile ducts	Korea	2016	Lee, T. Y., Cheon, Y. K., & Shim, C. S. (2016). Photodynamic therapy in patients with advanced hilar cholangiocarcinoma: percutaneous cholangioscopic versus peroral transpapillary approach. Photomedicine and Laser Surgery, 34(4), 150-156.
45	Bile ducts	USA	2011	Talreja, J. P., De Gaetani, M., Sauer, B. G., & Kahaleh, M. (2011). Photodynamic therapy for unresectable cholangiocarcinoma: contribution of single operator cholangioscopy for targeted treatment. Photochemical & Photobiological Sciences, 10(7), 1233-1238.
16	Bile ducts	UK	2002	Bown, S. G., Rogowska, A. Z., Whitelaw, D. E., Lees, W. R., Lovat, L. B., Ripley, P., ... & Hatfield, A. W. R. (2002). Photodynamic therapy for cancer of the pancreas. Gut, 50(4), 549-557.
36	Bile ducts	UK	2012	Pereira, S. P., Aithal, G. P., Ragunath, K., Devlin, J., Owen, F., & Meadows, H. (2012). Safety and long term efficacy of porfimer sodium photodynamic therapy in locally advanced biliary tract carcinoma. Photodiagnosis and photodynamic therapy, 9(4), 287-292.
232	Bile ducts	Korea	2012	Lee, T. Y., Cheon, Y. K., Shim, C. S., & Cho, Y. D. (2012). Photodynamic therapy prolongs metal stent patency in patients with unresectable hilar cholangiocarcinoma. World Journal of Gastroenterology: WJG, 18(39), 5589.
62	Bile ducts	China	2021	Li, Z., Jiang, X., Xiao, H., Chen, S., Zhu, W., Lu, H., ... & Zhang, D. (2021). Long-term results of ERCP-or PTCS-directed photodynamic therapy for unresectable hilar cholangiocarcinoma. Surgical Endoscopy, 35, 5655-5664.
39	Bile ducts	Russia	2018	Долгушин, Б. И., Сергеева, О. Н., Францев, Д. Ю., Кукушкин, А. В., Панов, В. О., Виршке, Э. Р., ... & Шишкина, Н. А. (2018). Внутрипротоковая фотодинамическая терапия при воротной холангиокарциноме у неоперабельных больных. Анналы хирургической гепатологии, 21(3), 106-118.

9	Lungs	Japan	2004	Okunaka, T., Kato, H., Tsutsui, H., Ishizumi, T., Ichinose, S., & Kuroiwa, Y. (2004). Photodynamic therapy for peripheral lung cancer. Lung Cancer, 43(1), 77-82.
30	Lungs	China	2013	Cai, X. J., Li, W. M., Zhang, L. Y., Wang, X. W., Luo, R. C., & Li, L. B. (2013). Photodynamic therapy for intractable bronchial lung cancer. Photodiagnosis and photodynamic therapy, 10(4), 672-676.
26	Lungs	Netherlands	1992	Sutedja, T., Baas, P., Stewart, F., & van Zandwijk, N. (1992). A pilot study of photodynamic therapy in patients with inoperable non-small cell lung cancer. European Journal of Cancer, 28(8-9), 1370-1373.
21	Lungs	UK	2003	Moghissi, K., & Dixon, K. (2003). Is bronchoscopic photodynamic therapy a therapeutic option in lung cancer?. European Respiratory Journal, 22(3), 535-541.
10	Lungs	California	2001	Jones, B. U., Helmy, M., Brenner, M., Serna, D. L., Williams, J., Chen, J. C., & Milliken, J. C. (2001). Photodynamic Therapy for Patients with Advanced Non–Small-Cell Carcinoma of the Lung. Clinical lung cancer, 3(1), 37-41.
12	Lungs	Japan	2015	Kimura, M., Miyajima, K., Kojika, M., Kono, T., & Kato, H. (2015). Photodynamic therapy (PDT) with chemotherapy for advanced lung cancer with airway stenosis. International journal of molecular sciences, 16(10), 25466-25475.
32	Lungs	Japan	2004	Kato, H., Harada, M., Ichinose, S., Usuda, J., Tsuchida, T., & Okunaka, T. (2004). Photodynamic therapy (PDT) of lung cancer: experience of the Tokyo Medical University. Photodiagnosis and photodynamic therapy, 1(1), 49-55.
21	Lungs	USA	1997	Cortese, D. A., Edell, E. S., & Kinsey, J. H. (1997, July). Photodynamic therapy for early stage squamous cell carcinoma of the lung. In Mayo Clinic Proceedings (Vol. 72, No. 7, pp. 595-602). Elsevier.
48	Lungs	Japan	1993	Hayata, Y., Kato, H., Konaka, C., & Okunaka, T. (1993). Photodynamic therapy (PDT) in early stage lung cancer. Lung cancer, 9(1-6), 287-293.
29	Lungs	Japan	1994	Imamura, S., Kusunoki, Y., Takifuji, N., Kudo, S., Matsui, K., Masuda, N., ... & Fukuoka, M. (1994). Photodynamic therapy and/or external beam radiation therapy for roentgenologically occult lung cancer. Cancer, 73(6), 1608-1614.

26	Lungs	Japan	1999	Okunaka, T., Hiyoshi, T., Furukawa, K., Yamamoto, H., Tsuchida, T., Usuda, J., ... & Kato, H. (1999). Lung cancers treated with photodynamic therapy and surgery. Diagnostic and therapeutic endoscopy, 5(3), 155-160.
165	Lungs	Japan	2007	Kato, H., Kawate, N., Kinoshita, K., Yamamoto, H., Furukawa, K., & Hayata, Y. (2007, September). Photodynamic therapy of early-stage lung cancer. In Ciba Foundation Symposium 146-Photosensitizing Compounds: Their Chemistry, Biology and Clinical Use: Photosensitizing Compounds: Their Chemistry, Biology and Clinical Use: Ciba Foundation Symposium 146 (pp. 183-197). Chichester, UK: John Wiley & Sons, Ltd..
29	Lungs	Japan	2008	Usuda, J. (2008). Photodynamic therapy for lung cancers based on novel photodynamic diagnosis using talaporfin sodium (NPe6) and autofluorescence bronchoscopy. Cancer Research, 68(9_Supplement), 1009-1009.
100	Lungs	UK	1999	Moghissi, K., Dixon, K., Stringer, M., Freeman, T., Thorpe, A., & Brown, S. (1999). The place of bronchoscopic photodynamic therapy in advanced unresectable lung cancer: experience of 100 cases. European journal of cardio-thoracic surgery, 15(1), 1-6.
160	Lungs	UK	2004	Moghissi, K., Dixon, K., Thorpe, J. A. C., Oxtoby, C., & Stringer, M. R. (2004). Photodynamic therapy (PDT) for lung cancer: the Yorkshire Laser Centre experience. Photodiagnosis and Photodynamic Therapy, 1(3), 253-262.
21	Lungs	UK	2007	Moghissi, K., Dixon, K., Thorpe, J. A. C., Stringer, M., & Oxtoby, C. (2007). Photodynamic therapy (PDT) in early central lung cancer: a treatment option for patients ineligible for surgical resection. Thorax, 62(5), 391-395.
36	Lungs	Japan	1992	Ono, R., Ikeda, S., & Suernasu, K. (1992). Hematoporphyrin derivative photodynamic therapy in roentgenographically occult carcinoma of the tracheobronchial tree. Cancer, 69(7), 1696-1701.
13	Lungs	USA	1992	Edell, E. S., & Cortese, D. A. (1992). Photodynamic therapy in the management of early superficial squamous cell carcinoma as an alternative to surgical resection. Chest, 102(5), 1319-1322.

29	Lungs	Japan	1994	Imamura, S., Kusunoki, Y., Takifuji, N., Kudo, S., Matsui, K., Masuda, N., ... & Fukuoka, M. (1994). Photodynamic therapy and/or external beam radiation therapy for roentgenologically occult lung cancer. Cancer, 73(6), 1608-1614.
22	Lungs	USA	1994	Lam, S. (1994, December). Photodynamic therapy of lung cancer. In Seminars in oncology (Vol. 21, No. 6 Suppl 15, pp. 15-19).
30	Lungs	Canada	1994	Sutedja, T., Lam, S., LeRiche, J. C., & Postmus, P. E. (1994). Response and pattern of failure after photodynamic therapy for intraluminal stage I lung cancer. Journal of Bronchology & Interventional Pulmonology, 1(4), 295-298.
12	Lungs	Germany	1996	Freitag, L., Korupp, A., Itzigehl, I., Dankwart, F., Tekolf, E., Reichle, G., ... & Macha, H. N. (1996). Experiences with fluorescence diagnosis and photodynamic therapy in a multimodality therapy concept of operated, recurrent bronchial carcinoma. Pneumologie (Stuttgart, Germany), 50(10), 693-699.
95	Lungs	Japan	1998	Kato, H. (1998). Photodynamic therapy for lung cancer—a review of 19 years' experience. Journal of Photochemistry and Photobiology B: Biology, 42(2), 96-99.
23	Lungs	Italy	1999	Patelli, M., Poletti, V., & Falcone, F. (1999). Photodynamic laser therapy for the treatment of early-stage bronchogenic carcinoma. Monaldi Archives for Chest Disease= Archivio Monaldi per le Malattie del Torace, 54(4), 315-318.
93	Lungs	Japan	2005	Furukawa, K., Kato, H., Konaka, C., Okunaka, T., Usuda, J., & Ebihara, Y. (2005). Locally recurrent central-type early stage lung cancer< 1.0 cm in diameter after complete remission by photodynamic therapy. Chest, 128(5), 3269-3275.
40	Lungs	Italy	2007	Corti, L., Toniolo, L., Boso, C., Colaut, F., Fiore, D., Muzzio, P. C., ... & Sotti, G. (2007). Long-term survival of patients treated with photodynamic therapy for carcinoma in situ and early non-small-cell lung carcinoma. Lasers in Surgery and Medicine: The Official Journal of the American Society for Laser Medicine and Surgery, 39(5), 394-402.
48	Lungs	Japan	2009	Endo C, Miyamoto A, Sakurada A, Aikawa H, Sagawa M, Sato M, Saito Y, Kondo T. Results of long-term follow-up of photodynamic therapy for roentgenographically occult bronchogenic squamous cell carcinoma. Chest 2009;136:369–375.

75	Lungs	Japan	2010	Usuda J, Ichinose S, Ishizumi T, Hayashi H, Ohtani K, Maehara S, Ono S, Honda H, Kajiwara N, Uchida O, et al. Outcome of photodynamic therapy using NPe6 for bronchogenic carcinomas in central airways >1.0 cm in diameter. Clin Cancer Res 2010;16:2198–2204.
64	Lungs	Japan	2010	Usuda J, Ichinose S, Ishizumi T, Hayashi H, Ohtani K, Maehara S, Ono S, Kajiwara N, Uchida O, Tsutsui H, et al. Management of multiple primary lung cancer in patients with centrally located early cancer lesions. J Thorac Oncol 2010;5:62–68.
41	Lungs	USA	2006	Ross P Jr, Grecula J, Bekaii-Saab T, Villalona-Calero M, Otterson G, Magro C. Incorporation of photodynamic therapy as an induction modality in non-small cell lung cancer. Lasers Surg Med 2006;38:881–889.
9	Lungs	California	2010	Weinberg BD, Allison RR, Sibata C, Parent T, Downie G. Results of combined photodynamic therapy (PDT) and high dose rate brachytherapy (HDR) in treatment of obstructive endobronchial non-small cell lung cancer (NSCLC). Photodiagn Photodyn Ther 2010;7:50–58.
10	Lungs	Korea	2013	Ji W, Yoo JW, Bae EK, Lee JH, Choi CM. The effect of Radachlorin® PDT in advanced NSCLC: a pilot study. Photodiagn Photodyn Ther 2013;10:120–126.Vie
20	Lungs	Russia	2013	Akopov AL, Rusanov AA, Molodtsova VP, Chistiakov IV, Kazakov NV, Urtenova MA, Rait M, Papaian GV. Photodynamic therapy in combined treatment of stage III non-small cell lung carcinoma [in Russian]. Khirurgiia (Mosk) 2013;3:17–20.
22	Lungs	Russia	2013	Akopov AL, Rusanov AA, Chistiakov IV, Urtenova MA, Kazakov NV, Gerasin AV, Papaian GV. Application of photodynamic therapy to reduce the amount of resection for non-small cell lung cancer [in Russian]. Vopr Onkol 2013;59:740–744.
21	Lungs	Russia	2014	Akopov A, Rusanov A, Gerasin A, Kazakov N, Urtenova M, Chistyakov I. Preoperative endobronchial photodynamic therapy improves resectability in initially irresectable (inoperable) locally advanced non small cell lung cancer. Photodiagn Photodyn Ther 2014;11:259–264
6	Stomach	Bulgaria	1991	Karanov, S., Shopova, M., & Getov, H. (1991). Photodynamic therapy in gastrointestinal cancer. Lasers in surgery and medicine, 11(5), 395-398.
22	Stomach	Germany	1998	Ell, C., Gossner, L., May, A., Schneider, H. T., Hahn, E. G., Stolte, M., & Sroka, R. (1998). Photodynamic ablation of early cancers of the stomach by means of mTHPC and laser irradiation: preliminary clinical experience. Gut, 43(3), 345-349.

386	Stomach	Japan	2016	Oinuma, T., Nakamura, T., & Nishiwaki, Y. (2016). Report on the National Survey of Photodynamic Therapy (PDT) for Gastric Cancer in Japan (a secondary publication). Laser therapy, 25(2), 87-98.
27	Stomach	Japan	1996	Mimura, S., Ito, Y., Nagayo, T., Ichii, M., Kato, H., Sakai, H., ... & Hayata, Y. (1996). Cooperative clinical trial of photodynamic therapy with photofrin II and excimer dye laser for early gastric cancer. Lasers in Surgery and Medicine: The Official Journal of the American Society for Laser Medicine and Surgery, 19(2), 168-172.
123	Stomach	France	1995	Sibille, A., Lambert, R., Souquet, J. C., Sabben, G., & Descos, F. (1995). Long-term survival after photodynamic therapy for esophageal cancer. Gastroenterology, 108(2), 337-344.
54	Stomach	France	1990	Patrice, T., Foultier, M. T., Yactayo, S., Adam, F., Galmiche, J. P., Douet, M. C., & Le Bodic, L. (1990). Endoscopic photodynamic therapy with hematoporphyrin derivative for primary treatment of gastrointestinal neoplasms in inoperable patients. Digestive diseases and sciences, 35, 545-552.
8	Stomach	Japan	2009	Nakamura, T., Ejiri, M., Fujisawa, T., Akiyama, H., Ejiri, K., Ishida, M., ... & Baba, S. (1990). Photodynamic therapy for early gastric cancer using a pulsed gold vapor laser. Journal of clinical laser medicine & surgery, 8(5), 63-67.
207	Stomach	China	1994	Jin, M., Yang, B., Zhang, W., & Wang, Y. (1994, March). Photodynamic therapy for upper gastrointestinal tumours over the past 10 years. In Seminars in surgical oncology (Vol. 10, No. 2, pp. 111-113). New York: John Wiley & Sons, Inc..
1	Stomach	Japan	2014	Nakamura, T., & Oinuma, T. (2014). Usefulness of photodynamic diagnosis and therapy using talaporfin sodium for an advanced-aged patient with inoperable gastric cancer (a secondary publication). Laser Therapy, 23(3), 201-210.
21	Stomach	Japan	2014	Namikawa, T., Inoue, K., Uemura, S., Shiga, M., Maeda, H., Kitagawa, H., ... & Hanazaki, K. (2014). Photodynamic diagnosis using 5-aminolevulinic acid during gastrectomy for gastric cancer. Journal of surgical oncology, 109(3), 213-217.
23	Stomach	UK	1995	Moghissi, K., Dixon, K., Hudson, E., & Stringer, M. (1995). Photodynamic therapy of oesophageal cancer. Lasers in medical science, 10, 67-71.

37	Stomach	USA	2006	Hahn, S. M., Fraker, D. L., Mick, R., Metz, J., Busch, T. M., Smith, D., ... & Glatstein, E. (2006). A phase II trial of intraperitoneal photodynamic therapy for patients with peritoneal carcinomatosis and sarcomatosis. Clinical cancer research, 12(8), 2517-2525.
215	Stomach	USA	2003	Litle, V. R., Luketich, J. D., Christie, N. A., Buenaventura, P. O., Alvelo-Rivera, M., McCaughan, J. S., ... & Fernando, H. C. (2003). Photodynamic therapy as palliation for esophageal cancer: experience in 215 patients. The Annals of thoracic surgery, 76(5), 1687-1693.
62	Stomach	Italy	2000	Corti, L., Skarlatos, J., Boso, C., Cardin, F., Kosma, L., Koukourakis, M. I., ... & Beroukas, K. (2000). Outcome of patients receiving photodynamic therapy for early esophageal cancer. International Journal of Radiation Oncology* Biology* Physics, 47(2), 419-424.
5	Brain	Germany	2013	Johansson A. C., Faber F., Kniebühler G., Stepp H., Sroka R., Egensperger R., Beyer W., Kreth F. W. Protoporphyrin IX Fluorescence and Photobleaching During Interstitial Photodynamic Therapy of Malignant Gliomas for Early Treatment Prognosis // Lasers in Surgery and Medicine. 2013. Vol. 45. No. 4. pp. 225-234.
25	Skin	Switzerland	2013	Piffaretti F., Zellweger M., Kasraee B., Barge J., Salomon D., van den BERGH H., Wagnières G. Correlation between Protoporphyrin IX Fluorescence Intensity, Photobleaching, Pain and Clinical Outcome of Actinic Keratosis Treated by Photodynamic Therapy // Dermatology. 2013. Vol. 227. No. 3. pp. 214-225.
19	Skin	USA	2014	Kanick S. C., Davis S. C., Zhao Y., Hasan T., Maytin E. V., Pogue B. W., Chapman M. S. Dual-channel red/blue fluorescence dosimetry with broadband reflectance spectroscopic correction measures protoporphyrin IX production during photodynamic therapy of actinic keratosis // Journal of Biomedical Optics. 2014. Vol. 19. No. 7. p. 75002
3	Brain	USA	2019	Ruiz A. J., LaRochelle E. P. M., Gunn J. R., Hull S. M., Hasan T., Chapman M. S., Pogue B. W. Smartphone fluorescence imager for quantitative dosimetry of protoporphyrin-IX-based photodynamic therapy in skin // Journal of Biomedical Optics. 2019. Vol. 25. No. 06. p. 1.

8	Lungs	USA	2017	Ong Y. H., Kim M. M., Finlay J. C., Dimofte A., Cengel K. A., Zhu T. C. Four-channel PDT dose dosimetry for pleural photodynamic therapy // Proceedings of SPIE - The International Society for Optical Engineering. 2017.
26	Skin	China	2021	Chen D., Wang Y., Zhao H., Haixia Qiu H. Q., Wang Y., Yang J., Ying Gu Y. G. Monitoring perfusion and oxygen saturation in port-wine stains during vascular targeted photodynamic therapy // Annals of Translational Medicine. 2021. Vol. 9. No. 3. p. 214.
